# Comparative Analysis of Rehabilitation Strategies Following Ankle Fracture Surgery: A Systematic Review

**DOI:** 10.7759/cureus.64315

**Published:** 2024-07-11

**Authors:** Abdullah Altuwairqi

**Affiliations:** 1 Orthopedic Surgery, King Abdulaziz University Faculty of Medicine, Jeddah, SAU

**Keywords:** quasi-randomized control trials, randomized control trials, rehabilitation strategies, ankle surgery, fracture, ankle

## Abstract

Considering various forms of immobilization that enable early weight-bearing or exercise initiation, rehabilitation following an ankle fracture can start shortly after the fracture has been repaired. Alternatively, after the period of immobility, physical or manual therapy may be used to begin rehabilitation. This systematic review aimed to compare different rehabilitation strategies after ankle fracture surgery. Four different databases (Scopus, Web of Science, PubMed/MEDLINE (Medical Literature Analysis and Retrieval System Online, and Google Scholar) were used to retrieve the relevant data using the Preferred Reporting Items for Systematic Reviews and Meta-Analyses (PRISMA) guidelines. Randomized and quasi-randomized controlled trials involving people undergoing every type of rehabilitation therapy following an ankle fracture surgery were taken into consideration. The main result was a limitation in activities. Adverse events and impairments were instances of such secondary outcomes. A total of 31 studies were found to be eligible for inclusion in this systematic review. The use of exercise and a removable form of immobilization during the immobilization phase to enhance activity limitation is supported by very little evidence. The patient's capacity to adhere to this treatment plan is crucial due to the possible higher risk. To support the available data, more carefully planned and sufficiently powered clinical trials must be conducted.

## Introduction and background

The most common type of fracture in the lower limbs is an ankle fracture [1]. Based on the number of malleoli involved, these fractures may be unimalleolar, bimalleolar, or trimalleolar [2]. The trimalleolar ankle fracture (TAF), a type of complicated ankle fracture, is comprised of a posterior malleolar fracture and a bimalleolar fracture. It is the rarest type, occurring in only 7% of ankle fractures [3].

Rehabilitation aims to regain the ankle’s strength, flexibility, and mobility without pain and with no possibility of future issues. Because ankle fractures are relatively intricate, and patients have different rehabilitation requirements, numerous rehabilitation approaches have been formed and applied in recent years [4]. Therefore, this systematic review will cover the wide range of rehabilitation interventions after ankle fracture surgery to establish the best approach that should be implemented to encourage the greatest degree of function and recovery.

Rehabilitation outcomes after ankle fracture surgery is a complex process that incorporates aspects of physiotherapy, and pharmacological management after ankle fracture surgery together with additional techniques like neuromuscular electrical stimulation or even robotic-assisted therapy among others [5]. The most common rehabilitation procedures are gradual weight-bearing exercises, joint mobilization for increasing the range of motion, and other exercises aimed at improving the function of the affected ankle [6]. In recent years, new technologies have emerged in the field of orthopedics and musculoskeletal systems as well and the knowledge about recovery has expanded to offer different approaches that can be used as additional or in combination with traditional treatment methods that might enhance the recovery time and final result. Some of these methods include the use of biological agents to enhance tissue healing, sophisticated bracing to enhance dynamic stability, and effective rehabilitation and physiotherapy programs to fit the patient’s needs and recovery progress [7].

There is insufficient data to support the effectiveness of interventions for rehabilitation following ankle fractures. According to a recent systematic review [8], there is little data to support the beneficial effects of rehabilitation therapies following surgical fixation for ankle fractures. The main purpose of this systematic review is to compare different rehabilitation strategies following ankle fracture surgeries.

## Review

Methodology

This systematic review was conducted according to the Preferred Reporting Items for Systemic Reviews and Meta-Analyses (PRISMA) guidelines [9] to compare different rehabilitation techniques used for ankle fractures after surgeries. The central question guiding this review was: Does the scientific evidence support any specific rehabilitation technique to be more effective than others?

Formulated in line with the PEOS strategy, the breakdown was as follows: P (population) referred to patients with ankle surgeries following rehabilitation therapy, E (exposure) denoted those who availed of rehabilitation treatment, O (outcome) explored the distribution patterns, and S (study type) randomized control trials and quasi-randomized control trials.

Search Strategy

A systematic search was done for the relevant literature on the following four databases to retrieve relevant studies: Scopus: ("rehabilitation strategies" OR "rehabilitation protocols" OR "rehabilitation programs") AND ("ankle fracture surgery" OR "ankle surgery") AND ("comparison" OR "effectiveness" OR "outcome" OR "results"), Web of Science: TS=("rehabilitation strategies" OR "rehabilitation protocols" OR "rehabilitation programs") AND TS=("ankle fracture surgery" OR "ankle surgery") AND TS=("comparison" OR "effectiveness" OR "outcome" OR "results"), PubMed/MEDLINE (Medical Literature Analysis and Retrieval System Online: ("rehabilitation strategies" OR "rehabilitation protocols" OR "rehabilitation programs") AND ("ankle fracture surgery" OR "ankle surgery") AND ("comparison" OR "effectiveness" OR "outcome" OR "results"), Google Scholar: "rehabilitation strategies" AND "ankle fracture surgery" AND "comparison" AND "effectiveness". Databases were also searched for published systematic reviews or ongoing systematic reviews on the same topic.

Studies Selection

During the identification of the articles, duplicates were removed by exporting them to EndNote Basic (EndNote, 2015; Clarivate Plc, Philadelphia, Pennsylvania, United States). Subsequently, the studies were chosen in two stages. Reviewer 1 evaluated titles and abstracts in duplicate, separately, throughout phase 1 to find studies that qualified.

All studies were chosen for inclusion upon the approval of a reviewer. When necessary, a second reviewer was invited in to help resolve any disagreements through group discussion. Therefore, abstracts and titles mentioning two things were considered acceptable: (i) rehabilitation strategies and (ii) ankle fracture surgery. To determine whether the publications had the relevant data for the systematic review, the articles were fully examined during the second evaluation step. We considered the following things as exclusion criteria: (i) lacking information regarding rehabilitation strategies, (ii) a case report, (iii) a narrative review study, (iv) a systematic review study, (v) a study based on an individual's judgment, and (vi) a study which is only based on differential diagnosis.

Data Collection

Data were separately extracted by the same reviewer from the chosen articles. Title, authors, name of journal, duration, kind of study, country, age, gender, number of participants, location, and rehabilitation strategy were noted for each included study. A Microsoft Excel spreadsheet (Microsoft Corporation, Redmond, Washington, United States) was used to extract and store data and records.

Results

Search Results

**Figure 1:**
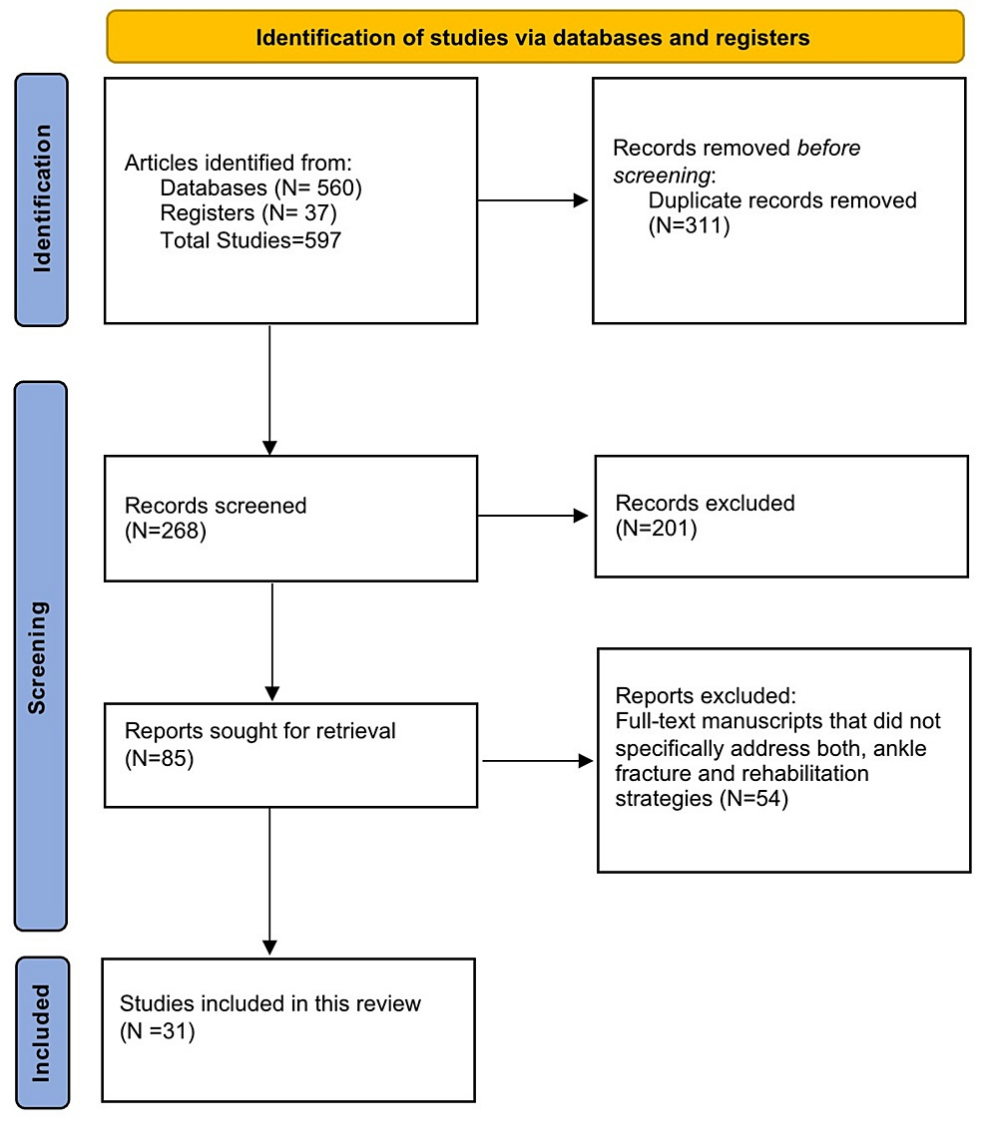
Studies selection using the guidelines of PRISMA PRISMA: Preferred Reporting Items for Systemic Reviews and Meta-Analyses

We found 597 studies using the criteria for selecting studies from four different databases, of which 311 were removed as duplicate records when the articles were sorted through EndNote software. After removing duplicates, we had 286 studies, which were all sorted for retrieval. Of these, 201 studies were not retrieved from the databases due to restricted access and were removed from inclusion in our study. After the remaining 85 full-text publications were reviewed for eligibility, 54 of them were rejected as these studies did not directly target focus on both, ankle fracture and rehabilitation strategy. Thus, this systematic review comprised 31 randomized control trials and quasi-randomized control trials (Figure 1).

Characteristics of Included Studies

The studies were first categorized according to when the interventions started (i.e., before, during, or after the immobilization period), and for those that started during the immobilization period, according to the type of orthopedic treatment.

Brink et al. conducted a study on patients with stable lateral malleolar fractures, comparing the use of an air-stirrup (N=33) with a DonJoy® orthosis (Enovis, Wilmington, Delaware, United States) (N=33) [10]. Their findings indicated that while both groups experienced similar recovery rates, those using the air-stirrup reported better comfort and mobility. Stuart et al. also examined the air-stirrup (N=20) but compared it with a walking cast (N=20) [11]. Walking cast shows lower promising results as compared to air-stirrup which is way more satisfactory.

Ginandes et al. evaluated the differences in outcomes between cast immobilization (N=6) and orthopedic follow-up (N=6); however, the study did not provide detailed information on the particular results [12]. Siddique et al. focused on patients with Weber B fractures, comparing no mobilization (N=22) with a plastic cast (N=22) [13]. According to their findings, the group that had cast immobilization responded much better in terms of strength and pain relief.

Comparing crepe and wool bandages with backslab, Reed et al. did not report any significant outcomes between these two groups [14]. Whitelaw et al. evaluated the use of a pneumatic walker (N=20) versus a plastic cast (N=20) [15]. While the recovery times for both groups were comparable, the pneumatic walker group reported better mobility and more satisfaction.

Ahl et al. conducted three different studies on weight-bearing protocols [16-18]. In 1986 and 1987, they compared immediate weight-bearing on the first day after surgery with delayed weight-bearing on the fourth day. Both studies concluded that there were no significant differences in healing between the groups, but those who began weight-bearing immediately reported greater comfort and faster return to function. Finsen et al. observed equivalent healing outcomes but higher satisfaction among patients with early weight-bearing after cast removal (N=19) when comparing weight-bearing with a plastic cast (N=19) [19].

The characteristics and comparison of rehabilitation strategies of the included studies are given in Table 1.

**Table 1:** Characteristics and comparison rehabilitation strategies of included studies

Reference	Year of Publication	Mean Age of Participants	Random Allocation	Concealed Allocation	Assessor binding	Dropouts <15%	Type of Fracture	Groups
Air-stirrup vs. other immobilization after orthopedic treatment								
[10]	1996	45 years	Yes	Yes	No	Yes	Stable lateral malleolar fracture	1. Air-stirrup (N.=33); 2. DonJoy orthosis (N.=33)
[11]	1989	Not reported by the study	Yes	Yes	No	Yes	(Weber A/B/C): 2/23/18	1. Air-stirrup (N=20); 2. Walking cast (N=20)
[12]	1999	Not reported by the study	Yes	No	Yes	Yes	Not reported by the study	1. Orthopedic Follow-up (N=6); 2. Cast Immobilization (N=6)
Cast Immobilization vs. No immobilization								
[13]	2005	Not reported by the study	No	No	Yes	No	All patient had Weber B	1. No Mobilization (N=22); 2. Plaster cast (N=22)
Type of Immobilization								
[20]	1998	40.8 years	Yes	Yes	No	No	(Weber A/B/C): 5/16/14	1. Crepe and wool bandage (N=27); 2. Backslab (N=28)
[15]	1991	Not reported by the study	No	No	No	No	Not reported by the study	1. Pneumatic Walker (N=20); 2. Plastic cast (N=20)
After surgery, compression stockings, and cast immobilization								
[21]	2014	35.2 years	Yes	No	Yes	Yes	Not reported by the study	1. Orthopedic Follow-up (N=31); 2. Cast Immobilization (N=31)
Weight-bearing after surgery								
[17]	1986	44 years	Yes	Yes	No	Yes	(Weber B/C):36/10	1. Plaster cast due to weight bearing on 1^st^ day after surgery (N=24); 2. Plaster cast due to weight bearing on 4th day after surgery (N=22)
[18]	1987	57 years	Yes	Yes	No	Yes	(Weber B/C):27/26	1. Plaster cast due to weight bearing on 1^st^ day after surgery (N=25); 2. Plaster cast due to weight bearing on 4th day after surgery (N=28)
[19]	1989	42 years	Yes	No	Yes	No	(uni-/bi-/trimalleolar): 24/10/22	1. Plaster cast, weight-bearing (N=19); 2. weight bearing after cast removal (N=19)
[22]	1996	36 years	No	No	No	Yes	(uni-/bi- or trimalleolar): 33/48	1. walking cast, physiotherapy (few) (N=41); 2. No immobilization (N=40)
[16]	1988	43 years	Yes	No	No	No	(Weber B/C): 24/17	1. Weight bearing (N=26); 2. ankle exercise and weight bearing (N=25)
[23]	1993	55 years	Yes	No	No	Yes	(supination -eversion IV: 28/6/6	1. weight bearing, ankle exercise (N=21); 2. Ankle exercise, Dorsal splint (N=19)
Exercise after surgery								
[24]	1999	42.7 years	Yes	Yes	Yes	Yes	bimalleolar	1. Backslab, ankle exercise, weight bearing (N=26); 2. Walking cast, backslab (N=26)
[25]	1986	36 years	Yes	No	No	No	(Weber A/B/C): 2/23/18	1. Ankle exercise, Backslab (N=20); 2. Plaster cast, physiotherapy after surgery (N=23)
[26]	1991	30.5 years	No	No	No	No	(uni-/bi- or trimalleolar): 18/17/9	1. Backslab (N=21); 2. Plaster cast (N=19)
[27]	1994	Not reported by the study	No	No	No	Yes	Not reported by the study	1. DonJoy orthosis and physiotherapy (N=30); 2. Plaster cast and physiotherapy (N=31)
[28]	2000	42.6 years	Yes	Yes	Yes	Yes	(supination -eversion IV: 28/6/6	1. Ankle exercise (N=27); 2. Fibreglass cast and physiotherapy (N=28)
[29]	1994	42.5 years	Yes	Yes	No	Yes	Not reported by the study	1. Ankle exercise, weight bearing (N=28); 2. Walking cast (N=25)
[30]	2003	41 years	Yes	Yes	Yes	Yes	(uni-/bi- or trimalleolar): 59/28/13	1. Air-stirrup, weight-bearing, ankle exercise (N=50); 2. Plaster cast (N=50)
[14]	2009	37.5 years	Yes	No	Yes	Yes	Weber C	1. weight bearing, orthosis (N=20); 2. Walking cast (N=20)
[14]	2009	36.4 years	Yes	Yes	Yes	Yes	Bimalleolar fracture	1. Pneumatic Brace (N=20); 2. Plaster cast (N=20)
[31]	2000	45 years	No	No	No	No	(Weber B/C): 27/13	1. Plaster cast, weight-bearing (N=20); 2. weight bearing (N=20)
[32]	1995	26 years	Yes	No	Yes	No	Not reported by the study	1. Ankle exercise (N=15); 2. Walking cast (N=15)
[33]	2007	36.1 years	No	No	No	Yes	Surgery Fracture (Weber B/C): 50/12	1. Fibreglass cast removable (N=33); 2. Fibreglass cast, Non-removable (N=29)
Exercise and weight bearing after surgery								
[34]	2007	40.3 years	Yes	Yes	No	Yes	(Weber A/B):c 1/44	1. Ankle exercise, weight bearing (N=23); 2. Plaster cast, partial weight bearing (N=22)
Electrotherapy after surgery								
[35]	1990	35 years	Yes	No	No	Yes	(uni-/bi- or trimalleolar):10/8/6	1. Ankle exercise (N=12); 2. After physiotherapy (N=12)
[36]	2005	42.5 years	Yes	Yes	Yes	Yes	Unimalleolar	1. removable braces, weight bearing (N=8); 2. removable braces, weight bearing (N=8)
[37]	2006	42.5 years	Yes	Yes	No	Yes	(uni-/bi- or trimalleolar):7/14/3	1. electrical muscles stimulation and weight bearing (N=12); 2. No electrical muscles stimulation and weight bearing (N=12)
Exercise and stretching after surgery								
[38]	2005	46.3 years	Yes	Yes	Yes	Yes	(Weber A/B/C/ missing): 29/91/15/15	1. 30 mins stretching and exercise/ day (N=51); 2. only exercise (N=50) 3. 6 mins stretching and exercise/day (N=49)
Manual therapy and exercise after surgery								
[39]	1991	44 years	Yes	Yes	Yes	No	(Weber B/C): 8/2	1. Manual therapy and exercise (N=7); 2. exercise only (N=5)

Laarhoven et al. investigate the walking cast with physiotherapy (N=41) versus no immobilization (N=40) in patients with uni-, bi-, or trimalleolar fractures [22]. Their results suggested that the walking cast group experienced better outcomes in terms of mobility and pain management. Ahl et al. explored weight-bearing alone (N=26) compared to ankle exercise and weight-bearing (N=25), noting improved functional outcomes in the latter group [16]. Similarly, when Ahl et al. compared ankle exercise with a dorsal splint (N=19) to weight-bearing plus ankle exercise (N=21), the former demonstrated a quicker functional recovery and a greater range of motion [23].

Dogra et al. conducted a study in 1999 and compared backslab, ankle exercise, and weight-bearing (N=26) with a walking cast and backslab (N=26), finding significantly better functional outcomes in the exercise group [24]. Egol et al. compared ankle exercise (N=27) with a fibreglass cast and physiotherapy (N=28) [28]. Their study demonstrated improved mobility and quicker return to normal activities for the exercise group. Lehtonen et al. investigated the use of an Air-stirrup with weight-bearing and ankle exercise (N=50) versus a plaster cast (N=50) [30]. The air-stirrup group showed enhanced functional recovery and patient satisfaction.

Ankle exercise and weight-bearing (N=23) with a plaster cast and partial weight-bearing (N=22) were compared by Honigmann et al., revealing that the exercise and weight-bearing group had faster functional recovery and better range of motion [34]. Christie and Willoughby looked into the benefits of ankle exercise (N=12) versus post-physiotherapy (N=12), but specific outcomes were not detailed [35]. A randomized control study by Handolin et al. compared the impact of removable braces with bearing weight (N=8) to the same with extra therapy (N=8), showing that the therapy group recovered more quickly and had stronger muscles [36].

When Hernandez et al. compared electrical muscle stimulation plus weight-bearing (N=12) to no stimulation and weight-bearing (N=12), they discovered that the stimulation group had greater muscle strength and recovered more quickly [37]. After comparing various stretching and exercise durations, Moseley et al. came to the conclusion that longer stretching sessions, 30 minutes, led to improved flexibility and function than shorter or no stretching sessions [38]. Furthermore, Wilson compared the effects of manual therapy plus exercise (N=7) versus exercise alone (N=5), finding that the manual therapy group had marginally better results in terms of joint mobility and pain reduction [39].

Discussion

A total of 31 randomized and quasi-randomized control studies were included in this review. The use of a removable form of immobilization in conjunction with exercise decreased activity limitation for rehabilitation interventions carried out during the period of immobilization following surgical fixation. Stretching, hypnosis, electrotherapy, manual therapy, and early or late weight-bearing did not appear to have an impact on activity limitation.

Studies generally showed no variation in adverse outcomes between groups, and the majority of adverse events were mild. This suggested that a variety of fracture severity levels may be treated safely using the majority of rehabilitation strategies. On the other hand, exercising throughout the immobilization time and employing a removable form of immobilization were linked to a higher rate of adverse outcomes. When implementing this intervention in clinical practice, a patient's capacity to safely follow the schedule of taking off immobilization, exercising, and reapplying immobilization is unquestionably a crucial consideration.

Activity limitation was the key outcome we used to assess the efficacy of rehabilitation because we were primarily interested in changes in function (i.e., a patient-oriented outcome) than in disability. Only five of the 21 studies that examined activity limitation had enough information to determine the extent of the treatment impact, but nine of them revealed statistically significant differences in activity limitation between treatment and control interventions. In every one of these investigations, exercise was examined while patients were immobilized following surgical fixation.

The Olerud Molander Ankle Score was employed in three studies [29,33,40], and after the treatment and follow-up periods, the treatment impact seemed to be clinically significant. After the six-week therapy, Egol et al. [28] employed a grading system developed by Mazur et al. [41] and observed a statistically significant treatment effect for the use of a removable kind of immobilization and exercise. Because Losch et al. employed a dichotomous variable and this study's treatment details are unknown, the results can only be interpreted as preliminary [14]. Collectively, these trials imply that the combination of exercise with a removable form of immobilization may result in clinically significant improvements in activity limitation. This has to be strengthened with additional research and balanced against any adverse effects.

The current findings are in line with a recent systematic review [42] that found some evidence to suggest starting exercise when immobilized. By assessing the evidence for additional interventions and offering quantitative data, this review strengthens the existing body of research. However, there are several limitations to the information included in this review. First off, in most of the included studies, the results reported were based on individual research due to clinical and statistical heterogeneity between studies, which precluded meta-analyses, and the minimal number of papers available for some comparisons. Second, the lack of power in some of the studies may have affected the small sample size.

## Conclusions

The use of exercise and a removable form of immobilization during the immobilization phase to enhance activity limitation is supported by very little evidence. The patient's capacity to adhere to this treatment plan is crucial due to the possible higher risk. To support the available data, more carefully planned and sufficiently powered clinical trials must be conducted.
